# Dual optofluidic distributed feedback dye lasers for multiplexed biosensing applications

**DOI:** 10.1038/s41598-023-42671-4

**Published:** 2023-10-06

**Authors:** Tyler Sano, Ravipa Losakul, Holger Schmidt

**Affiliations:** Department of Electrical and Computer Engineering, University of California Santa Cruz (UCSC), 1156 High Street, Santa Cruz, CA 95064 USA

**Keywords:** Optics and photonics, Integrated optics

## Abstract

Integrated optofluidic devices have become subjects of high interest for rapid biosensor devices due to their unique ability to combine the fluidic processing of small volumes of microfluidics with the analysis capabilities of photonic structures. By integrating dynamically reconfigurable optofluidic lasers on-chip, complex coupling can be eliminated while further increasing the capabilities of sensors to detect an increasing number of target biomarkers. Here, we report a polydimethylsiloxane-based device with two on-chip fluidic distributed feedback (DFB) laser cavities that are integrated with an orthogonal analyte channel for multiplexed fluorescence excitation. One DFB grating is filled with 4-(dicyanomethylene)-2-methyl-6-(4-dimethylaminostyryl)-4*H*-pyran dissolved in dimethyl sulfoxide. The second grating is filled with rhodamine 6G dissolved in a diluted ethylene glycol solution. We present characterization of both lasers through analysis of the lasing spectra for spectral narrowing along with a power series to observe threshold behavior. We then demonstrate simultaneous detection of two different fluorescent microbeads as a proof of concept for scalable, single biomarker analysis using on-chip optofluidic lasers.

## Introduction

In the wake of the recent SARS-CoV-2 pandemic, the onset of a “tridemic” describes the simultaneous presence and mass spread of influenza, COVID-19, and respiratory syncytial virus^1^. The necessity to diagnose several different diseases further deepens the need for rapid biosensors with multiplexed detection capabilities. Optofluidic technologies are being widely utilized in biosensor devices in order to integrate optical components and waveguiding with the manipulation of small volumes of fluidic samples^2–8^. Several optofluidic biosensors have been demonstrated on varying platforms, primarily silicon^9,10^ and polydimethylsiloxane (PDMS)^11^. PDMS provides several unique advantages for biosensor platforms due to its optical transparency^12^, refractive index manipulability^13^, biocompatibility^14^, mechanical elasticity^15^, and ease of fabrication^16^ which enables rapid prototyping of device designs^17–19^. A primary component that PDMS optofluidics enables is the addition of on-chip fluidic lasers. Two main benefits that on-chip lasers offer in the development of biosensors are adding a versatile and reconfigurable light source while simultaneously facilitating a more simplified alignment of excitation sources.

Single molecule detection capabilities on optofluidic waveguide devices have been demonstrated using an external laser that has been fiber-coupled orthogonally to a fluidic analyte channel via a solid-core optical waveguide^20,21^. Multiplexed sensing of multiple biomarkers was implemented by coupling multiple lasers into a single mode optical fiber, which was then coupled to a device containing a multimode interference waveguide^22^. This method encodes spectral information of fluorescently tagged biomarkers in wavelength-dependent multimode interference excitation profiles. The fiber-to-chip coupling can prove to be difficult and time consuming as well as be prone to damaging the fiber or chip facets from abrasive contact. Some optofluidic devices aim to reduce this risk by directly implanting optical fibers within devices^23^. This issue can be entirely resolved by using on-chip light sources, which dramatically reduces alignment difficulty by integrating the excitation laser monolithically into the planar chip architecture^24^.

PDMS optofluidics have been used to construct various laser structures including ring resonators^25^, Fabry–Perot cavities^26,27^, droplet arrays^28^, random lasers^29^, and distributed feedback (DFB) cavities^30^. Optofluidic-based DFB dye lasers have been shown to be particularly effective in achieving single mode lasing while maintaining low threshold pump powers^30–32^. Optofluidics are very powerful in the development of on-chip laser systems due to their versatility. By exchanging gain media or reconfiguring the cavity dimensions, the lasing wavelength can be tuned^33^. Here, we present spatial multiplexing as an alternative through the extension of a DFB laser chip that uses corrugated PDMS sidewalls^24^, by including a second DFB Bragg grating for introduction of a second excitation wavelength. By keeping the excitation spots neatly separated, spatially multiplexed fluorescence signals can be easily analyzed. Additionally, the DFB lasers can be tuned and modulated independently. The DFB grating geometry is space efficient and compatible with a multiple pump laser scheme, enabling scalability through the addition of more gratings into the device architecture in both in-plane and out-of-plane axes^34^. We demonstrate simultaneous detection of two different fluorescent microspheres as a proxy for two separately labeled biomarkers. In principle, the DFB array could be extended through the addition of more DFB gratings with variation in grating periods as well as gain media to achieve scalable multiplexed biosensing.

## Methods

### Device design

**Figure 1:**
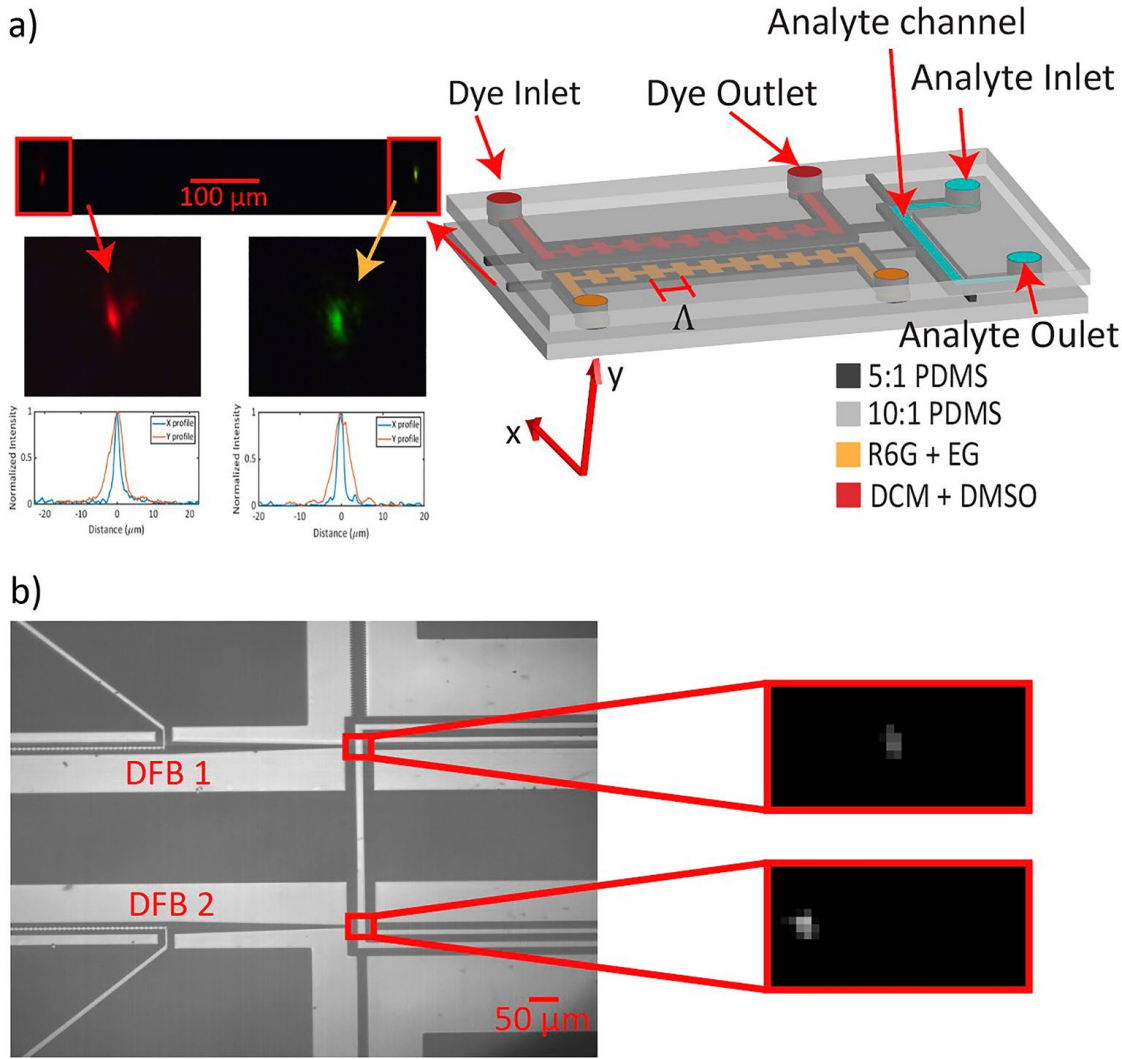
Device design. (**a**) Chip design with two parallel DFB gratings with grating period Λ = 8 µm. One grating is used as the rhodamine 6G laser and the other is used as the DCM laser. (Gratings are enlarged to enhance visibility). The camera image on the left is the laser modes coupled into free space using a 10× objective. Zoomed mode images were collected using a 40× objective and displayed with x and y mode profiles. (**b**) Top-down camera image showing the intersection of the DFB lasers with an orthogonal analyte channel. The two zoomed images show examples of fluorescence detection of a red fluorescent bead (top) and a flash red fluorescent bead (bottom).

The dual DFB chip shown in Fig. 1a is composed entirely of PDMS and features two parallel liquid-core distributed feedback Bragg gratings. The grating is formed by sidewall corrugations, which induce alternating effective refractive indices. Wavelength selection is enabled by choosing design parameters following the Bragg condition:

1 $$m{\lambda }_{m}=2{n}_{eff}\Lambda ,$$

where $m$ is the Bragg order, ${\lambda }_{m}$ is the resonant wavelength corresponding to $m$, ${n}_{eff}$ is the effective index of the waveguide mode, and $\Lambda$ is the grating period.

Here, we use 4 mm long DFB gratings with a periodicity of 8 µm and corrugation depth of 2.5 µm. Output laser emissions from the DFB gratings are coupled on either side by solid-core waveguides. Optical waveguiding is achieved through the solid-core and liquid-core waveguides via total internal reflection index guiding. By varying the base-to-curing agent mixing ratio of PDMS, higher and lower refractive index PDMS can be made^13^. In this device, we use 5:1 PDMS as the high index guiding media and 10:1 PDMS as the lower index cladding. The 5:1 PDMS solid-core waveguides have a refractive index of 1.4032 at 574.6 nm and 1.4003 at 656.5 nm and are surrounded horizontally by air gaps and vertically by 10:1 PDMS cladding with a refractive index of 1.3960 at 574.6 nm and 1.3929 at 656.5 nm. All liquid core channels are filled with vacuum tubes applied at the outlet of each channel. Dye solvents were carefully chosen to ensure a higher refractive index than the 5:1 PDMS gratings as well as the 10:1 cladding. The first DFB grating is filled with 5 mM 4-(Dicyanomethylene)-2-methyl-6-(4-dimethylaminostyryl)-4*H*-pyran (DCM) dissolved in dimethyl sulfoxide (DMSO), which has a refractive index of 1.4739. Using DCM as a laser dye is particularly effective due to its property of having a large Stokes shift. DMSO is selected as the solvent for DCM to maximize the quantum yield of DCM^35^. The second DFB grating is filled with 5 mM rhodamine 6G dissolved in a solution of 85% ethylene glycol and 15% deionized water with a refractive index of 1.4147. The respective Bragg orders are $m=34$ and $m=39$ for the DCM and rhodamine 6G lasers. The fabrication of our device follows standard soft lithography procedures^16^. Briefly, photolithography is used to produce an SU-8 pattern on a silicon wafer. This master is used as a mold, where PDMS is spun on to generate a thin waveguide layer. Thicker cladding layers are poured on top of the waveguide layer as well as on a blank silicon wafer to act as a capping layer. The waveguide layer is then pealed and bonded to the capping layer using an oxygen plasma treatment.

An enlarged camera image illustrating the intersection of the DFB laser emissions with the analyte channel is shown in Fig. 1b. The DFB lasers are coupled to the analyte via the tapered solid-core waveguide that narrows from 25 to 5 µm over a 300 µm distance. The tapered waveguide allows for the maximum collection of DFB emission and optimal excitation in the analyte channel with a single mode. The two red rectangles indicate the regions of interest, with the camera images on the right showing examples of detected fluorescence events from commercially obtained fluorescent microspheres. 1 µm red fluorescent beads (FluoSphere, Invitrogen, Waltham, MA USA) were used as the analyte corresponding to the rhodamine 6G laser, seen in the top image, with a peak excitation of 580 nm and peak emission of 605 nm. The bottom image shows an example detection from a 2 µm flash red microsphere (Bangs Laboratories, Fishers, IN USA), which was used as the analyte corresponding to the DCM laser with a peak excitation of 660 nm and a peak emission of 690 nm.

### Experimental setup

**Figure 2:**
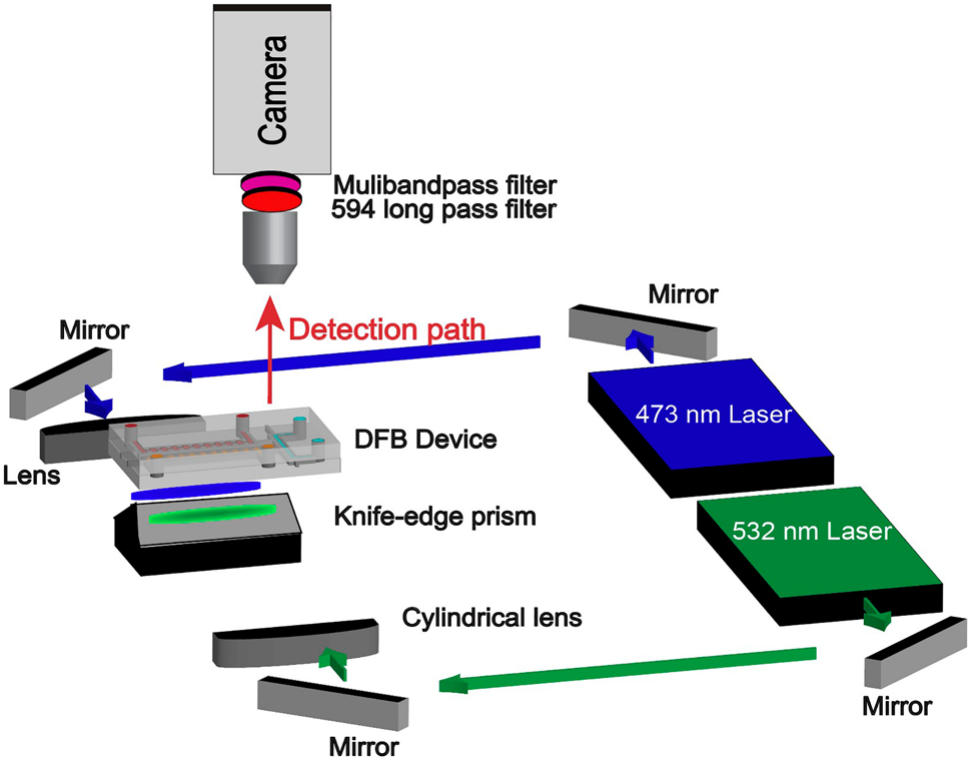
Experimental setup of the dual DFB pumping scheme. The 532 nm pump laser used to pump the R6G laser is aligned through a cylindrical lens to elongate the pump beam. This aids in optimizing the pump power sent through the gain medium. The 473 nm pump laser used to pump the DCM laser passes through a lens to shape the beam along the grating. Fluorescence emission is collected by a camera aligned above the device with fluorescence filters to remove erroneous signal from the pump and DFB lasers.

The experimental setup is illustrated in Fig. 2. The DFB device is mounted on two parallel glass slides with a gap below the two gratings. Vacuum lines are connected to the outlets of the DFB and analyte channels to pull fluids through the chip. The chip is then suspended directly above a knife-edge prism. The prism allows for symmetric optical paths to hit a 45° mirror and form a bottom-up pumping scheme. The 532 nm pulsed pump laser (TEEM Photonics STG-03E-140, Meylan, France) is passed through a cylindrical lens to shape the beam into a focused ellipse to optimally pump the DFB cavity. Similarly, the 473 nm pulsed pump laser (RPMC Lasers Inc. SB1 473-3-5, O’Fallon, MO USA) is passed through a spherical lens since the output of the microchip laser is already elliptical in shape. Both optical paths can also use an additional cylindrical lens to control the length of the pump beam. A camera (Andor Zyla, Oxford Technologies, Belfast, UK) is aligned above the chip to aid with alignment of the pump lasers as well as record video traces of the regions of interest in the analyte channel which will later be post processed into fluorescence time domain traces. A 594 nm long pass fluorescence filter and a 440-521-607-694-809 multiband pass filter are placed in the camera path during fluorescence measurements to filter out the pump lasers as well as the emission from the DFB dye lasers, leaving only the fluorescence emission to be collected by the camera. The camera collection of fluorescence events contrasts with typical collection using a single photon counter aligned to the solid-core waveguides on-axis with the analyte channel. While inherently less sensitive, camera detection allows for simplified alignment and convenient event validation via analysis of the recorded video. Additionally, for conducting multiplexed sensing experiments with single mode excitation, camera detection drastically reduces the complexity of capturing signals since the fluorescence events do not need to pass through a dichroic filter and then be recoupled into their respective single photon counters.

## Results

The on-chip optofluidic dye lasers were characterized for thresholds by analyzing the output power as a function of average input power. Output power was measured by cropping the fundamental mode seen in the camera image in Fig. 1a and aligning into a power meter. A line was fit to the two linear regions indicative of below and above threshold, with the intersection of the two lines taken to be the threshold input power.

**Figure 3:**
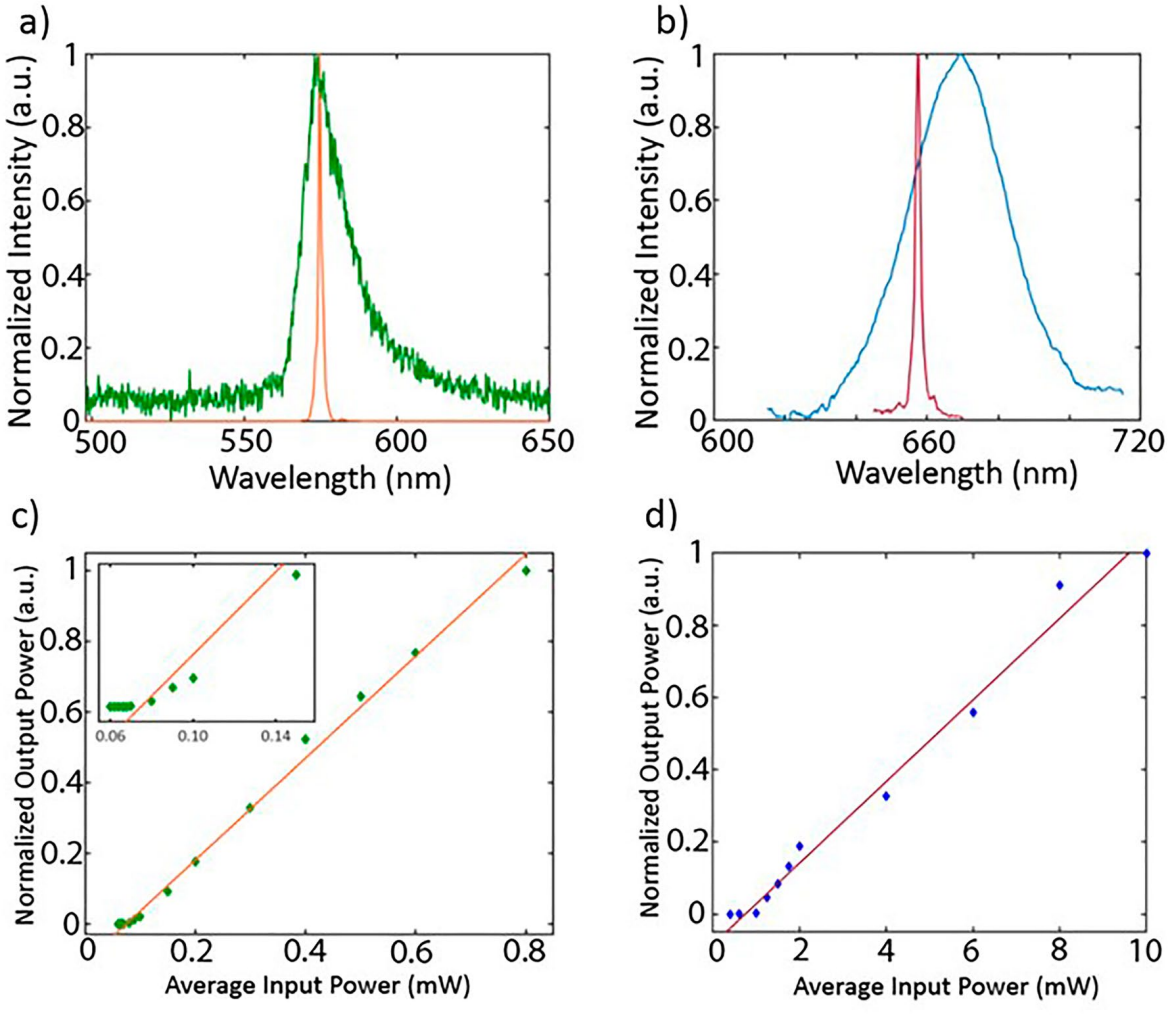
Laser characterization. (**a**) A broadband rhodamine 6G amplified spontaneous emission (ASE) spectrum (green) is shown below threshold with a lasing spectrum (orange) shown when pumped above threshold. (**b**) Broadband DCM ASE spectrum (blue) shown below threshold with a lasing spectrum (red) overlayed when pumped above threshold. (**c**) Normalized average output power as a function of input power for the rhodamine 6G laser with a linear fit over the above threshold region (orange). (**d**) Normalized average output power as a function of input power for the DCM laser with a linear fit over the above threshold region (dark red).

The power series that are shown in Fig. 3c,d illustrate clear threshold behavior with two linear regimes. The threshold average input power for the rhodamine 6G laser is 87.9 µW, which corresponds to a threshold fluence of 52.7 mW/cm^2^. The DCM DFB laser had a threshold average input power of 0.77 mW, which corresponds to a fluence of 307.6 mW/cm^2^. The outputs of the DFB lasers were then fiber-coupled one-by-one and put into an optical spectrum analyzer (Yokogawa AQ6374, Newman, GA USA). The amplified spontaneous emission spectrum (ASE) of rhodamine 6G was taken below threshold with an average input pump power of 70 µW, while the lasing spectrum was taken above threshold at an average input pump power of 1.3 mW (Fig. 3a). The pump power of the 473 nm laser was set to 0.7 mW to observe the ASE spectrum of DCM. The pump power was then increased to 2 mW to capture the lasing spectrum of the DCM DFB laser (Fig. 3b). Both lasing spectra demonstrate evident spectral narrowing when pumped above threshold. The rhodamine 6G lasing spectrum peaked at a central lasing wavelength of 574.6 nm and had a full width at half maximum (FWHM) of 1.24 nm, compared to the 656.5 nm central wavelength and 1.73 nm FWHM of the DCM laser.

### Fluorescent bead detection

The primary goal of this work was to demonstrate the multiplexed sensing capabilities of the dual DFB laser device. Two different types of fluorescent microspheres were excited using the on-chip DFB lasers in order to determine the ability to sense two fluorescent analytes with high specificity. As stated above, red fluorescent beads were used as the target for the rhodamine 6G laser, while flash red beads were used as the target for the DCM laser.

The chip was first aligned such that both pump lasers aligned under their respective DFB gratings. For the fluorescence detection, the 532 nm pump laser was operated at 3 mW and the 473 nm pump laser was operated at 10 mW to ensure that both DFB lasers were operating well above threshold. The first grating was then filled with the 5 mM rhodamine 6G in diluted ethylene glycol solution by placing a 3 µL drop of the solution on top of the inlet. Similarly, the second grating was then filled with the 5 mM DCM dissolved in DMSO. A solution of $2\times {10}^{6}$ beads/mL flash red beads and $4 \times {10}^{7}$ beads/mL red beads was made and 3 µL of the solution was placed above the inlet of the analyte channel and pulled through the device using a constant vacuum pressure of − 60 kPa applied to the outlet. Once the solution filled the analyte channel, the camera window was reduced to the analyte channel, enabling a reduced exposure time of 5 ms. The 200 frames per second frame rate ensures a high time resolution. A 5-min recording was then started, which is capped by the data transfer rate between camera and computer.

**Figure 4:**
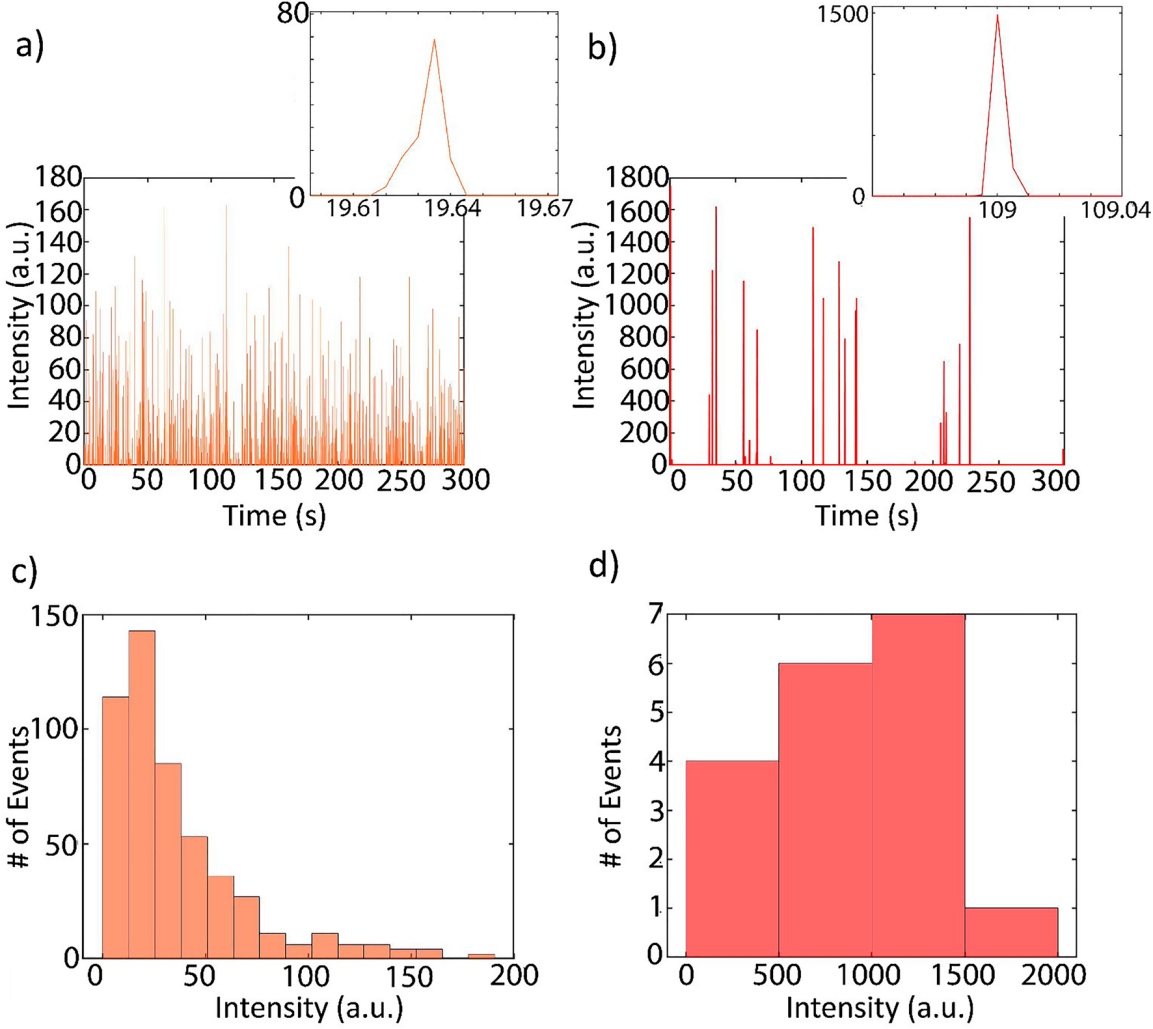
Simultaneous dual fluorescent microsphere detection. (**a**) Fluorescence time domain trace from the rhodamine 6G laser region of interest illustrating peaks from detection of 1 µm red fluorescent beads. The zoomed image shows individual event. (**b**) Fluorescence time domain trace from the DCM laser excitation region of interest illustrating peaks from detection of 2 µm flash red fluorescent beads. (**c**) Intensity histogram of the red bead events from the rhodamine 6G excitation region. (**d**) Intensity histograms flash red bead events from the DCM excitation region.

After the recording was completed and the file was exported, the two regions of interest shown in Fig. 1b were cropped and the pixel intensities were integrated for each region of interest and each frame. Since each frame corresponds to 5 ms of time, the pixel intensities were then plotted in the time domain. Fig. 4a,b illustrate the fluorescence time domain traces collected from the simultaneous detection of red and flash red fluorescent microspheres.

Analysis was carried out using a parallel cluster wavelet analysis (PCWA) algorithm^36^. In this application, a Ricker wavelet, defined as the normalized negative second derivative of a Gaussian function, was used in a continuous wavelet transform as it closely resembles the expected signals resulting from single mode excitation. The continuous wavelet transform was calculated according to Eq. (2). By convolving the fluorescence time domain trace, $f(t)$, with the Ricker wavelet, $\psi (t)$, across a $\Delta t$ range, the PCWA algorithm is able to efficiently detect particles apart from noise while extracting information about each event’s FWHM in the time domain as well as its peak height for analysis of the signal intensity distribution.

2 $$C\left(t,\Delta t\right)=\langle f,{\psi }_{t,\Delta t}\rangle ={\int }_{-\infty }^{\infty }f\left({t}^{\prime}\right)\frac{1}{\sqrt{\Delta t}}{\psi }^{*}\left(\frac{t-{t}^{\prime}}{\Delta t}\right)d{t}^{\prime}.$$

Concentration of beads were determined using Eq. (3), where $N$ is the total number of events, ${V}_{exc}$ is the excitation volume determined by the vertical and horizontal FWHM of the single excitation mode (determined by imaging of the fundamental mode coupled to the solid-core waveguide) multiplied by the width of the analyte channel, $T$ is the total length of the collected time trace, and $\Delta {t}_{avg}$ is the average FWHM of the detected events in the time domain.

3 $$c=\frac{N{V}_{exc}}{T{\mathrm{\Delta t}}_{avg}}.$$

The excitation volume was calculated to be 4.37 × 10^–10^ mL, resulting from a mode width and height of 4.1 µm and 7.1 µm, and a channel width of 15 µm, respectively. For the shown time domain trace, there were 448 fluorescent events detected in the rhodamine 6G excitation region with a $\Delta {t}_{avg}$ of 11.6 ms. This corresponds to a detected concentration of $c=3.97 \times {10}^{7}$ beads/mL. This concentration corresponds to a molar concentration of 65.9 fM. Conversely, the DCM excitation region produced 18 events with a $\Delta {t}_{avg}$ of 10.7 ms, corresponding to a detected concentration of $c=1.47 \times {10}^{6}$ beads/mL. The equivalent molar concentration is 2.4 fM. Thus, the detected concentrations demonstrate good agreement with the expected fluorescent bead concentrations. The signal heights can be plotted in the histograms illustrated in Fig. 4c,d. The signal intensity from each fluorescent microsphere is dependent upon the location of the microsphere in the excitation mode as well as the speed of the bead flowing through the excitation region^37^. Higher intensity signals typically come from slower particles that move closer to the channel walls and spend a longer time in the excitation region, while lower intensity signals tend to come from faster particles that spend less time in the excitation region. For the rhodamine 6G signals, we observe a peak and an upper tail of a Gaussian distribution. The lack of a lower tail indicates that any missed events are likely of lower signal intensities, however the close concentration match suggests that the number of missed particles is low. The DCM signal distribution resembles a rough Gaussian distribution, but the lack of abundant signals prevents the distribution from being well defined.

**Figure 5:**
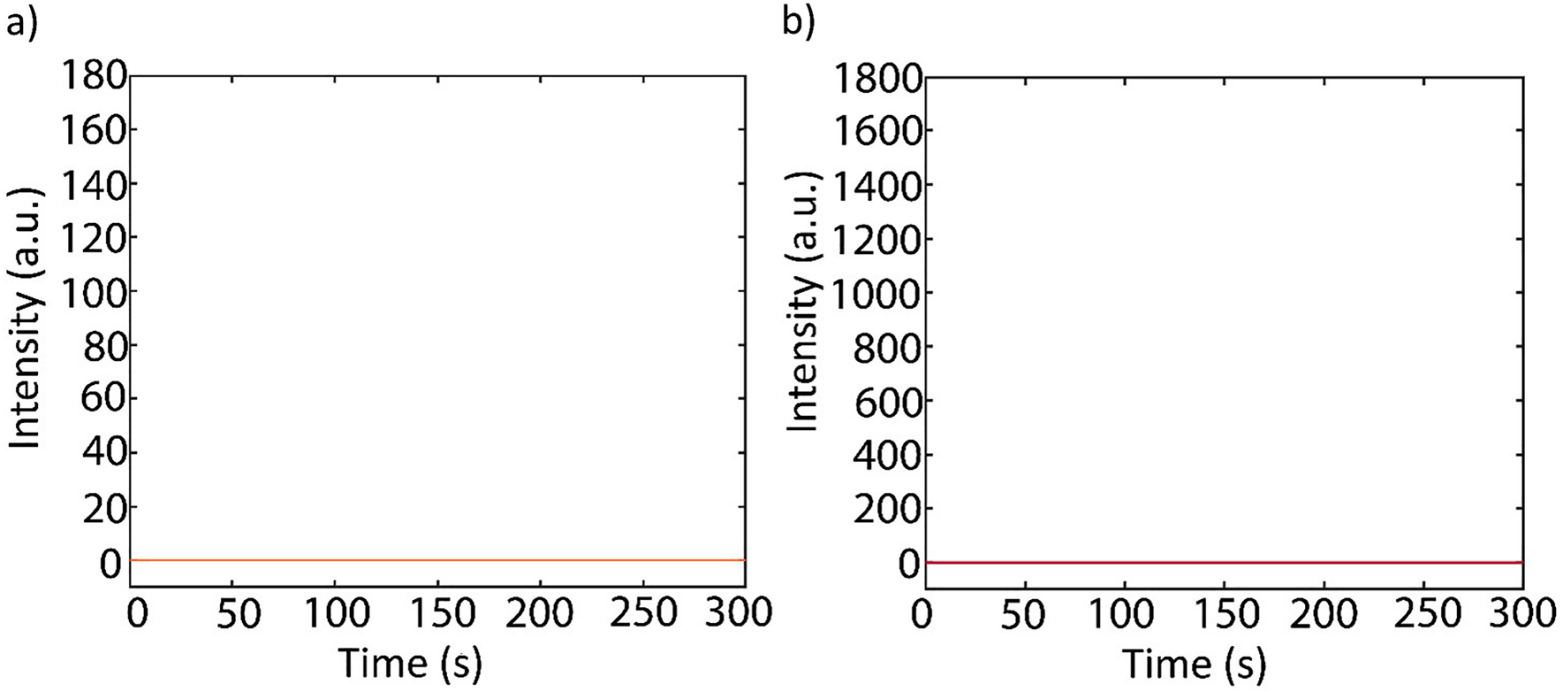
Negative controls. (**a**) Fluorescence time domain trace from R6G excitation region of interest illustrating no false events from 2 µm flash red beads. (**b**) Fluorescence time domain trace from DCM excitation region of interest demonstrating no false events from 1 µm red beads.

Negative controls were run to validate the specificity of detection from each laser (Fig. 5). The presence of fluorescence signals would indicate that the detection is not wavelength specific. The negative control for the rhodamine 6G DFB laser consisted of flowing only flash red fluorescent microspheres with the DCM DFB laser turned off and the rhodamine 6G laser turned on. As illustrated in Fig. 5a, the fluorescence time domain trace is void of any detected events. Similarly, Fig. 5b shows the fluorescence time domain trace as a result of flowing only red fluorescent microspheres with the DCM DFB laser turned on and the rhodamine 6G laser turned off. The absence of false positive signals in the negative controls illustrates simultaneous specific detection of two different fluorescent microspheres at femtomolar concentrations.

## Discussion

We have demonstrated simultaneous detection of two different fluorescent microspheres using two parallel optofluidic DFB lasers, representing a concrete example of a PDMS optofluidic platform that is capable of scalable multiplexing with additional DFB gratings and a variety of gain media. By integrating multiple on-chip DFB lasers with fluorescence biomarker detection, meticulous and tedious fiber alignment systems can be eliminated without losing the capability of carrying out complex multiplexed sensing experiments. This results in simpler, free space pumping of on-chip lasers that are directly integrated and coupled to fluidic analyte channels. Future steps towards increasing the number of DFB lasers on-chip could lead to scalable multiplexing experiments using different dyes and to enable more lasing wavelengths using the same dye. Mixing multiple dyes into a single DFB grating while using a color camera for detection could also expand multiplexing capabilities. While finite space exists for additional pump lasers and coupling optics, several strategies can be implemented to scale up the number of on-chip sources and multiplex sensing levels. We can expand the utility of a single pump laser by using it to pump additional DFB lasers that are filled with the same dye but have different gratings periods. We can also take advantage of using laser dyes that can be pumped with the same wavelength but emit at different wavelengths. Multiple excitation wavelengths can also be combined with other multiplexing methods such as velocity multiplexing^38^, where different biomarkers with the same fluorescent tag flow through parallel fluidic channels at different flow rates to generate different $\Delta {t}_{avg}$ values. Furthermore, these laser arrays can be integrated with a previously demonstrated all-in-one optofluidic biosensor that used a single DFB laser that had rhodamine 6G as its fluorescent dye to detect Zika nucleic acids using a bead-based sandwich assay completed on-chip^39^. This work provides progress towards the development of a rapid biosensor with the potential to aid in prevention or reduction in severity of future epidemics as well as control and differentiate between various infections using target-specific fluorescent biomarker tagging.

## Data Availability

The data presented in this study are available upon request from the corresponding author.
